# Biofabrication of Zinc Oxide Nanoparticles With *Syzygium aromaticum* Flower Buds Extract and Finding Its Novel Application in Controlling the Growth and Mycotoxins of *Fusarium graminearum*

**DOI:** 10.3389/fmicb.2019.01244

**Published:** 2019-06-12

**Authors:** Thimappa Ramachandrappa Lakshmeesha, Naveen Kumar Kalagatur, Venkataramana Mudili, Chakrabhavi Dhananjaya Mohan, Shobith Rangappa, Bangari Daruka Prasad, Bagepalli Shivaram Ashwini, Abeer Hashem, Abdulaziz A. Alqarawi, Jahangir Ahmad Malik, Elsayed Fathi Abd_Allah, Vijai Kumar Gupta, Chandra Nayaka Siddaiah, Siddapura Ramachandrappa Niranjana

**Affiliations:** ^1^Department of Studies in Biotechnology, University of Mysore, Mysore, India; ^2^Microbiology Division, Defence Food Research Laboratory, Mysore, India; ^3^Toxicology and Immunology Division, DRDO-BU-Centre for Life Sciences, Bharathiar University, Coimbatore, India; ^4^Department of Studies in Molecular Biology, University of Mysore, Mysore, India; ^5^Adichunchanagiri Institute for Molecular Medicine, Mandya, India; ^6^Department of Physics, BMS Institute of Technology and Management, Bengaluru, India; ^7^East Point College of Medical Sciences and Research Centre, Bengaluru, India; ^8^Botany and Microbiology Department, College of Science, King Saud University, Riyadh, Saudi Arabia; ^9^Mycology and Plant Disease Survey Department, Plant Pathology Research Institute, Agriculture Research Center (ARC), Giza, Egypt; ^10^Department of Plant Production, College of Food and Agricultural Sciences, King Saud University, Riyadh, Saudi Arabia; ^11^Department of Chemistry and Biotechnology, Tallinn University of Technology, Tallinn, Estonia

**Keywords:** green synthesis, zinc oxide nanoparticles, mycotoxin, deoxynivalenol, zearalenone

## Abstract

*Fusarium graminearum* is a leading plant pathogen that causes Fusarium head blight, stalk rot, and Gibberella ear rot diseases in cereals and posing the immense threat to the microbiological safety of the food. Herein, we report the green synthesis of zinc oxide nanoparticles from *Syzygium aromaticum* (SaZnO NPs) flower bud extract by combustion method and investigated their application for controlling of growth and mycotoxins of *F. graminearum*. Formation of SaZnO NPs was confirmed by spectroscopic methods. The electron microscopic (SEM and TEM) analysis revealed the formation of triangular and hexagonal shaped SaZnO NPs with size range 30–40 nm. The synthesized SaZnO NPs reduced the growth and production of deoxynivalenol and zearalenone of *F. graminearum* in broth culture. Further analysis revealed that treatment of mycelia with SaZnO NPs resulted in the accumulation of ROS in the dose-dependent manner. Also, SaZnO NPs treatment enhanced lipid peroxidation, depleted ergosterol content, and caused detrimental damage to the membrane integrity of fungi. Moreover, SEM observations revealed that the presence of diverged micro-morphology (wrinkled, rough and shrank surface) in the macroconidia treated with SaZnO NPs. Taken together, SaZnO NPs may find a potential application in agriculture and food industries due to their potent antifungal activity.

## Introduction

The world population is estimated to reach about 10.5 billion by 2050, and accessibility of food needs to rise about 60% to accomplish the food security (Alexandratos and Bruinsma, 2012). The food security could be improved through production and minimizing the food losses. In the interim, agriculture is facing climate change and limitation in land and water, and therefore, hard to increase food production (Aulakh and Regmi, 2013). As per FAO, about 1300 million tons of food are wasted per annum worldwide due to inappropriate post-harvesting practices (FAO, 2012). Thus, saving the food loss at a post-harvesting session could be the greatest resolve to improve the food security (Gustavsson et al., 2011). In this context, particularly fungal infestations are the foremost accountable for food loss, and FAO estimated that about 25% of agricultural commodities were contaminated with fungi (Smith et al., 2016). The fungal infestation brings unacceptable features in color, texture, flavor, and taste of food and introduces hazardous mycotoxins in food (Kumar et al., 2016).

The major mycotoxigenic fungal species liable for infestations are *Aspergillus*, *Alternaria*, *Claviceps*, *Fusarium*, *Penicillium*, and *Stachybotrys* (Richard, 2007). Among these mycotoxigenic species, *Fusarium graminearum* is one of the most accountable for food waste and food safety worldwide. The *F. graminearum* is devastating plant pathogen and causes FHB, stalk rot, and Gibberella ear rot diseases in cereals and accountable for loss of billion dollars worldwide (Pasquali et al., 2016). It is also liable for production of hazardous mycotoxins, including DON, NIV, and ZEA (Mudili et al., 2014). These mycotoxins classified as the group 3 carcinogens and cause a variety of toxic effects, such as neurotoxicity, hepatotoxicity, immunotoxicity, reproductive and developmental toxicity, nephrotoxicity, etc. (Richard, 2007; Venkataramana et al., 2014; Escrivá et al., 2015; Kalagatur et al., 2017, 2018b). Consequently, *F. graminearum* is posing the immense threat to the microbiological safety of food. Since last decade, microbiologists and food technologists have given greatest attention toward *F. graminearum* infestations and proposed variety of combat methods (Kalagatur et al., 2015, 2018c,d,e; Kumar et al., 2016; Sellamani et al., 2016). Unfortunately, most of the approaches have limitations and not acceptable. The application of synthetic fungicidal agents is not satisfactory due to its hazardous nature and development of fungicide-resistant fungi. Though, irradiation and high-pressure processing decontamination techniques are still not wide-spread owing to the prerequisite of high-cost equipment and skilled workers. The plant-based antifungal materials, i.e., crude extracts, essential oils, and phytocompounds are biodegradable and not cause side-effects (George et al., 2016; Kalagatur et al., 2018d,e). Therefore, there is a huge demand for the plant-based antifungals. Hence, plant-based synthesis of antifungal nanoparticles could be the novel, promising, and satisfactory tactic.

At present, nanotechnology has created an enormous revolution in the researchers. Zinc oxide nanoparticles (ZnO NPs) have roused a considerable interest among scientists due to their environment-friendly and potential applications in the field nanomedicine, biosensors, antibacterial, antifungal, and photochemical activities (Zhang P. et al., 2017; Zheng et al., 2017; Zhu L. et al., 2017). ZnO is a semiconductor material (II–VI), having a wide band gap of *E*_g_ = 3.37 eV semiconductor material with an extensive exciton binding energy of 60 meV at room temperature (Iorgu et al., 2013). In recent years, the interest in ZnO NPs applications for microbial control have grown considerably due to development of multidrug resistance among microbes (Kadiyala et al., 2018). Green synthesis of ZnO NPs is preferred over chemical and physical method as it is an eco-friendly and cost-effective method without use of high temperature, pressure toxic chemicals, and eliminates the generation of hazardous substances (Basnet et al., 2018). Among the green mediated synthesis of ZnO NPs, plant-mediated biofabrication of ZnO NPs has gained popularity over another method as its easily available in large quantity, it contains secondary metabolites, it reduces the processing time in maintaining bacterial and fungal cultures and cross-contamination is negligible among plant extracts (Ahmed et al., 2017; Vijayakumar et al., 2017).

The *Syzygium aromaticum* L. (clove) is an evergreen tree that rises to a height ranging from 7 to 13 m, which produces a flower bud that has numerous medicinal properties. The main constituents of the clove buds are eugenol, carvacrol, thymol, and cinnamaldehyde (Chen et al., 2016). The health benefits of cloves have been known for centuries such as an effective remedy for a headache, indigestion problem, cough, nausea, hypertension, etc. (Kheawfu et al., 2017). Clove buds extracts have been used in Ayurveda as a source of antimicrobial agents against oral microorganisms that are generally connected with dental caries and also finds use in fragrance and flavoring industries (Hamed et al., 2012; Chatterjee and Bhattacharjee, 2015).

To the best of our knowledge, for the first time an attempt has been made to report the inhibition of *F. graminearum* growth and mycotoxins production from SaZnO NPs, synthesized from *S. aromaticum* flower bud extracts, no such study has been reported, so far.

## Materials and Methods

### Chemicals and Reagents

Zinc nitrate hexahydrate (Zn (NO_3_)_2_.6H_2_O), DON (purity 98% TLC and CAS number: 51481-10-8), Dulbecco’s phosphate buffer saline pH 7.4 (DPBS), lipid peroxidation assay kit, and ZEA (purity 99% HPLC and CAS number: 17924-92-4) were purchased from Sigma-Aldrich, India. Peptone, Tween 80, CZA, CZB, PI, sterile gauze, DCFH-DA, syringe filters (0.45 μm), double-layered muslin cloth, and Whatman no.1 filter papers were obtained from HiMedia, India. Immuno-affinity columns specific for DON and ZEA were obtained from Vicam, Waters, United States. All other chemicals and reagents were of AR grade and purchased from Merck Millipore, India. The plastic ware was purchased from Nunc, India. Glassware used were washed with 2% sodium hypochlorite solution and maintained in sterile condition.

### Preparation of *Syzygium aromaticum* Extract

*Syzygium aromaticum* flower buds were collected from the local market, Mysore, and the voucher was identified and safeguarded in Department of studies in Biotechnology, University of Mysore, India.

Flower buds (10 g) were rinsed 2–3 times in de-ionized water, shade dried for 144 h and powdered utilizing a clean electric blender and put away in sterile polyethylene test sacks before utilize. The powder obtained was macerated with solvent hexane at the ratio of 1:6 (w/v) in a glass screw cap reagent bottle and was kept for 96 h. Subsequently, the supernatant was filtered through twofold layered muslin fabric, and the supernatant was recovered by centrifugation at 4000 rpm for 10 min. The supernatant was sieved through Whatman No. 1 filter paper and achieved filtrate was considered as mother extract and kept aseptically in a darker screw capped bottle at 4°C for additionally utilize (Cansian et al., 2016).

### GC-MS Analysis of *S. aromaticum* Extract

To determine chemical constituents of *S. aromaticum* flower bud, the extract which served as a mother extract was subjected to gas chromatography (GC) and mass spectrophotometer (MS) analyses using Clarus 680 GC equipped with Elite-5MS (95% dimethylpolysiloxane, 5% biphenyl, ID × 250 μm df, 30 m × 0.25 mm) and the components were separated using Helium gas as a transporter gas at a steady flow of 1 mL/min. A quantity of 1 μL *S. aromaticum* extract was injected into the instrument, and the oven temperature was maintained as per the following: 60°C for 2 min and elevated till it reaches 300°C at the rate of 10°C min^-1^ where it was seized for 6 min. Mass detector conditions were as follows: ionization mode electron impact at 70 eV and ion source temperature 240°C with a scan interval of 0.1 to 0.2 s. The spectrums of the obtained constituents were equated with the database of known spectrum constituents from the GC-MS NIST (2008) library (Bhuiyan, 2012; Ali et al., 2014).

### Biosynthesis of SaZnO NPs

Zinc nitrate (Zn(NO_3_)_2_6H_2_O) was used as the substrate, and *S. aromaticum* extract was used as fuel at the ratio of 1:5 (*w/v*) and mixed to form a solution. The solution was transferred to China dish and mixed in magnetic stirrer for ∼ 5–10 min and then placed in a preheated muffle furnace maintained at 400 ± 10°C. The entire combustion process was completed in less than 4 min (Lakshmeesha et al., 2014). The final product as-synthesized SaZnO NPs was off-white color.

### Characterization of SaZnO NPs

UV-Vis spectroscopic absorption measurements were carried out at room temperature using a UV–Vis spectrophotometer Beckman Coulter, (DU739, Germany) over the range of 200–800 nm. Fourier transform infrared (FTIR) transmittance was carried out with a PerkinElmer Spectrum 1000, (Shimadzu-8400S) in the range of 450–4000 cm^-1^. The crystallinity and phase purity of SaZnO NPs were characterized by XRD using a Rigaku Desktop MiniFlex II X-ray powder diffractometer with Cu kα radiation at an angle 2θ (λ = 0.15418 nm). The particle size was determined by Scherrer equation.

$$\text{D=}\frac{\text{0}.\text{89}\lambda }{\beta \cos\theta }$$

Where λ is the wavelength (Cu Kα) of X-Rays, β is the full width at half- maximum (FWHM) of the peak, and θ is the diffraction angle. The XRD pattern of SaZnO NPs was analyzed with the ICDD Powder Diffraction File database (International Centre for Diffraction Data) using Crystallographica Search-Match Version 2, 1, 1, 1. The surface morphology of SaZnO NPs samples is studied using SEM HITACHI (S-3400 N, Japan). The size of SaZnO NPs was studied using TEM (Tecnai G2 Spirit Bio-TWIN Transmission Electron Microscope).

### Antifungal and Antimycotoxin Activity of SaZnO NPs on *F. graminearum*

#### Fungi Cultural Conditions

*Fusarium graminearum* isolated from maize kernels in our previous study (Mudili et al., 2014) were grown for 14 days at 28°C in CZA plates. The fungal spores were collected in peptone water containing 0.01% Tween 80 and mycelium debris was separated from the suspension by filtering through sterile gauze. The spore number was estimated by hemocytometer and fixed approximately to 10^6^ spores/mL and used in further studies.

#### Treatment of SaZnO NPs

Different concentrations of SaZnO NPs (25, 50, 75, 100, 125, and 140 μg/mL) were added to 250 mL of Erlenmeyer flask that contained 100 mL of sterile CZB. The flasks were inoculated with 10 μL of fungal spore suspension (10^6^ spores/mL) and incubated at 28°C for 14 days with 12 h of light per day. The flasks not contain SaZnO NPs and inoculated with fungi was considered as a control. Following the incubation period, the fungal mycelium was separated from the broth by sieving through Whatman no.1 filter paper. The fungal mycelium and filtrate were used for determination of fungal biomass (growth) and mycotoxins, respectively.

#### Determination of Fungal Growth (Biomass)

Subsequently, attained fungal mycelium was washed with deionized water for twice and packed in pre-weighed Whatman no.1 filter paper. The sample was subjected to drying at 60°C employing hot-air oven and weighed.

#### Quantification of Mycotoxins

Briefly, the attained filtrate was blended with acetonitrile (1:1, v/v) at 120 rpm for 30 min and 15 mL of the blend was discretely passed through immunoaffinity columns of DON and ZEA mycotoxins at speed of 3 – 4 drops per sec as per the guidance of the manufacturer, Vicam, United States. Following, mycotoxins were eluted in 5 mL of acetonitrile and dried out at 60°C using a water bath. Next, the final residue was re-dissolved in 1 mL of acetonitrile and used for detection of DON and ZEA mycotoxins. The quantification of DON and ZEA was done using HPLC (Shimadzu, Japan) equipped with C18 column (5 μm, 250 × 4.6 mm) and a fluorescence detector. The analysis was carried out in reverse phase, and mobile phase was acetonitrile and water (1:1, v/v) with a flow rate of 1 mL/min, and an injection volume of sample was 25 μL. The excitation and emission of the detector were set to 365 and 455 nm for DON, and 334 and 450 nm for ZEA, respectively. The quantification of DON and ZEA was determined from their corresponding standard calibration curves. For the construction of calibration curves, stock solutions of DON and ZEA were prepared separately in acetonitrile (1 mg/mL) and different concentrations were made in distilled water and used for HPLC quantification. The retention time and limit of detection for DON and ZEA were noticed as 7.31 and 12.06 min, and 24 and 21 ng/mL, respectively. The calibration curves of mycotoxins were constructed with peak area versus concentration.

### Assessment of Antifungal Mechanism of SaZnO NPs on *F. graminearum*

The antifungal mechanism of SaZnO NPs on *F. graminearum* was assessed by determining the intracellular ROS, lipid peroxidation, ergosterol content, membrane integrity, and micromorphology of macroconidia.

#### Estimation of ROS Generation

A quantity of 10 μL of fungal spore suspension was aseptically inoculated to 1 mL of CZB in 12-well plate and allowed to grow for 3 days at 28°C. Following, different concentration of SaZnO NPs (25, 50, 75, 100, 125, and 140 μg/mL) was added and further incubated at 160 rpm (rotary shaker) and 28°C for 24 h with 12 h light per day. The CZB contained only fungal inoculum and not treated SaZnO NPs was referred as a control. Following, samples were stained with 5 μM of DCFH-DA and washed for twice with DPBS. The optical density was measured at excitation of 495 nm and emission of 550 nm using the plate reader (Synergy H1, BioTek, United States) and results was expressed in percentage of ROS release with respect to control. The standard curve of ROS released versus hydrogen peroxide was constructed and used for quantification of ROS release. In addition, phase-contrast and fluorescent images were also captured using the inverted fluorescence microscope (EVOS, Thermo Scientific, United States).

#### Estimation of Lipid Peroxidation

A quantity of 1 mL CZB was inoculated with 10 μL of fungal spore suspension (10^6^ spores/mL) in 12-well plate and incubated at 28°C for 3 days. Following, fungi were treated with different doses of SaZnO NPs (25, 50, 75, 100, 125, and 140 μg/mL) and incubated at 160 rpm (rotary shaker) and 28°C for 24 h with 12 h light. The fungal sample not treated with SaZnO NPs was denoted as a control. The MDA, the end product of lipid peroxidation was quantified using lipid-peroxidation assay kit as per instructions from the manufacturer (Sigma-Aldrich). The results were expressed with respect to control.

#### Estimation of Ergosterol Content

A volume of 10 μL of fungal spore suspension (10^6^ spores/mL) and different concentration of SaZnO NPs (25, 50, 75, 100, 125, and 140 μg/mL) were added to 100 mL of CZB in 250 mL Erlenmeyer flask and incubated at 28°C and 160 rpm (rotary shaker) for 7 days with 12 h light per day. Following, 50 mg of fungal mycelia was recovered and washed with distilled water for twice and used for determination of ergosterol. The extraction and HPLC quantification of ergosterol were done as per our previous reported methodology of Sellamani et al. (2016).

#### Assessment of Membrane Integrity of Spores

A quantity of 1 mL of seven-day-old fungal spore suspension (10^6^/mL) was treated with different concentration of SaZnO NPs (25, 50, 75, 100, 125, and 140 μg/mL) in 2 mL Eppendorf and incubated at 160 rpm (rotary shaker) and 28°C for 24 h with 12 h of light. The spore suspension not treated with SaZnO NPs was considered as control. Following spore suspension was stained with 5 μM of PI for 15 min and washed for twice with DPBS by centrifugation at 5000 rpm for 5 min. The PI stained spores were measured at excitation of 490 nm and emission of 635 nm using the flow cytometry (Beckman Coulter) and results was expressed in percentage with respect to control. The phase-contrast and fluorescent images of spores were captured under an inverted fluorescence microscope (EVOS, Thermo Scientific, United States).

#### Observation of Micromorphology of Macroconidia

The fungi were grown at 28°C for 7 days with 12 h light per day on CZA plate and 1 cm^2^ of mycelia was collected under aseptic condition. The CZA slides were prepared with different concentrations of SaZnO NPs (100, 125, and 140 μg/mL) and inoculated with 7-days old fungal mycelium (1 cm^2^) and incubated in the sterile humid atmospheric chamber at 28°C for 3 days with 12 h light per day. The CZA slide not contains SaZnO NPs and inoculated with fungi was control. Following, mycelium mat was recovered and attached to dual adhesive carbon tape and micrographs of macroconidia were captured under SEM (FEI Quanta 200, United States) in an environmental mode at 20 KV.

### Statistical Analysis

The studies were performed independently for six times, and results were expressed as mean ± SD. The data were analyzed by one-way ANOVA and statistical difference between experimental groups was compared by Tukey’s test using GraphPad Prism trial version 7. The *p*-value was stated as a significant at ≤ 0.05.

## Results

### GC-MS of *S. aromaticum* Extract and Characterization of SaZnO NPs

**FIGURE 1:**
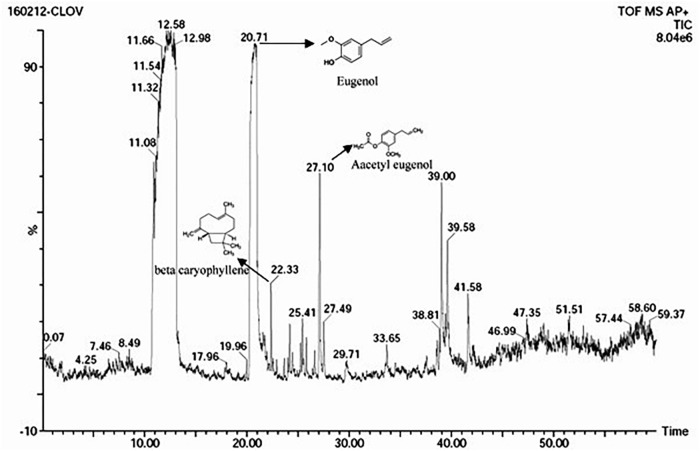
GC-MS chromatogram of *S. aromaticum* flower buds hexane extract.

**FIGURE 2:**
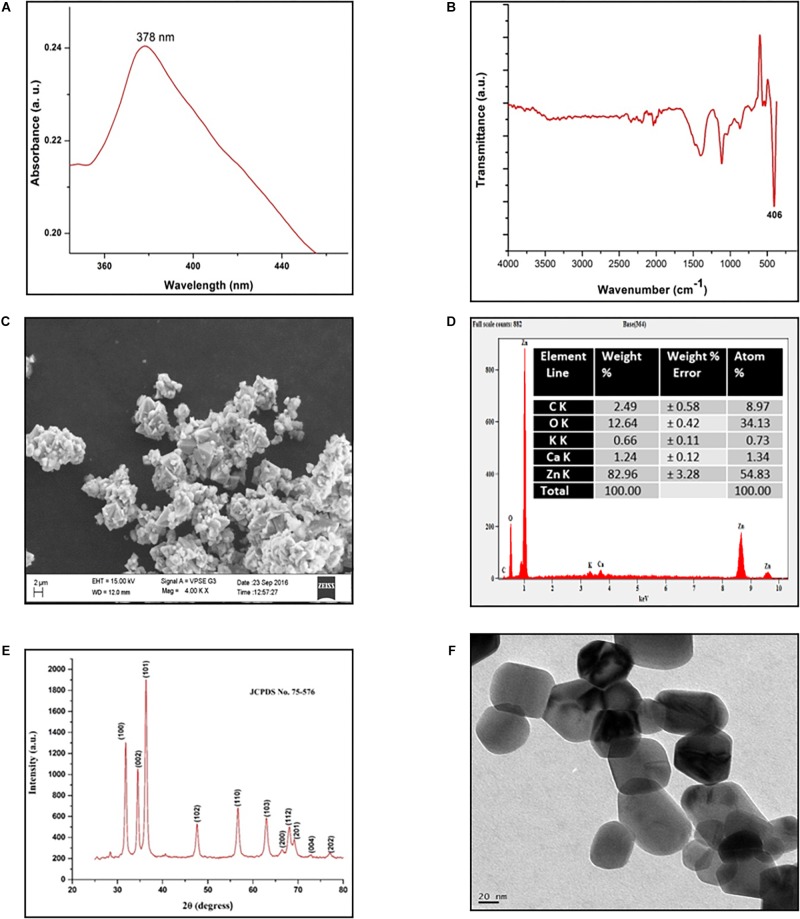
Characterization of SaZnO-NPs. **(A)** UV-Visible spectrum of SaZnO NPs, **(B)** FTIR spectrum of SaZnO NPs, **(C)** SEM micrograph of SaZnO NPs, **(D)** EDX pattern of SaZnO NPs **(E)** XRD pattern of SaZnO NPs, **(F)** TEM micrograph of SaZnO NPs.

The *S. aromaticum* hexane extract subjected for GC-MS revealed the presence of eugenol (C_10_H_10_O_2_), beta-caryophyllene (C_15_H_24_), acetyl eugenol (C_12_H_14_O_3_), and many other compound types (Figure 1 and Table 1). The results were in agreement with the earlier studies (Ali et al., 2014; Sheweita et al., 2016; Chen et al., 2017a,b). Eugenol was regarded as the major component of *S. aromaticum* (Kovács et al., 2016), the probable mechanism of formation of SaZnO NPs may be attributed to repeating structural units of eugenol participating in intermolecular cross-linking with ZnO to form a stable SaZnO NPs structure. UV–visible spectra of SaZnO NPs are shown in Figure 2A. It can be observed that there is a maximum absorption peak in the UV region of ∼378 nm, which is a characteristic band for the wurtzite hexagonal pure SaZnO (Reddy et al., 2011). Figure 2B shows the FTIR spectrum of SaZnO NPs acquired in the range of 400–4000 cm^-1^. The appearance of peaks in the region of 406 cm^-1^ is ascribed to the formation of metal-oxygen bond (Kavyashree et al., 2015). The SEM micrographs (Figure 2C) shows the SaZnO NPs formed are of highly agglomerated, to form a well-defined triangular to nearly hexagonal shape in nature, which can be attributed to wurtzite structure of ZnO NPs which is in accordance with recent findings (Velmurugan et al., 2016; Ahmed et al., 2017).

**Table 1:** Major components of *S. aromaticum* flower buds hexane extract.

Component	Eugenol	Beta caryophyllene	Acetyl eugenol
Retention time	20.71	22.33	27.10
Molecular formula	C_10_H_10_O_2_	C_15_H_24_	C_12_H_14_O_3_
Molecular weight	164.2	204.36	206.24


The EDX (Figure 2D) study was carried out for the synthesized SaZnO NPs showed the peaks corresponded to zinc yield (82.96%) and oxygen elements (12.64%) of the ZnO NPs which indicates that the SaZnO NPs synthesized is of pure form (Chen et al., 2017b). The XRD pattern of the synthesized SaZnO NPs as seen in Figure 2E. The appearance of all diffraction peaks corresponds to (100), (002), (101), (102), (110), (103), (200), (112), (201), (004), (202), (104), and (203) planes are indicated to hexagonal wurtzite structure of ZnO and could be readily indexed to (JCPDS Card No. 75-576). The Miller indices (100), (002), and (101) XRD peaks correspond to Bragg angles 31.8, 34.5, and 36.4°, respectively (Reddy et al., 2011) which indicates that the ZnO NPs are of good crystalline in nature. The (hkl) standard intensity plane was taken from JCPDS data. All the diffraction peaks can be fit to the hexagonal wurtzite phase ZnO with cell constants of *a* = 3.25 A° and *c* = 5.21 A° (Suresh et al., 2015). No other additional diffraction peaks from impurities were observed in the spectrum confirms the phase purity of ZnO NPs. The average grain size (D) of ZnO samples calculated from the Scherrer’s equation (Prashanth et al., 2018) using the sharpest reflection was 35.69 nm. The TEM image (Figure 2F) supported the SEM results of SaZnO NPs which are hexagonal in shape and having an average size in the range of 30–40 nm.

### Effect of SaZnO NPs on Fungal Growth and Mycotoxins Production of *F. graminearum*

**FIGURE 3:**
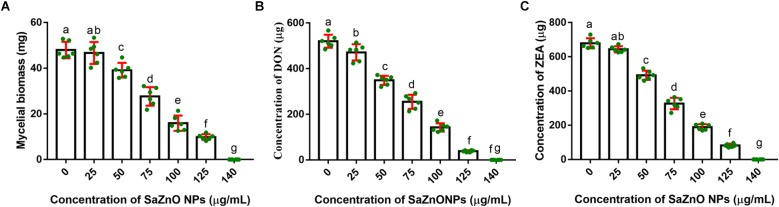
Dose-dependent inhibitory effect of SaZnO NPs on **(A)** mycelial biomass (fungal growth), **(B)** deoxynivalenol (DON), and **(C)** zearalenone (ZEA) of *F. graminearum* in broth culture. The data was analyzed by one-way ANOVA according to Tukey’s multiple comparison test and experimental clusters denoted with different alphabetic letters were significant (*p* < 0.05).

In the present study, the inhibitory effect of SaZnO NPs on fungal growth (fungal biomass) and mycotoxins (DON and ZEA) production of *F. graminearum* was determined in broth culture. The SaZnO NPs has successfully decreased the fungus growth and mycotoxins production in broth culture (Figure 3). A quantity of 47.98 ± 3.61 mg of fungal biomass, 520.5 ± 28.46 μg of DON, and 678.8 ± 29.40 of ZEA were noticed in 100 mL of control broth culture (SaZnO NPs untreated). Whereas, the SaZnO NPs treated test samples were exhibited the lower levels of fungal growth and mycotoxins compared to control. The complete eliminations of fungal growth and mycotoxins were noticed at 140 μg/mL of SaZnO NPs. The inhibitory effects of SaZnO NPs on fungal growth and mycotoxins were assessed by constructing linear regression curves (Supplementary Figure S1). The linear regression curves have exhibited goodness of fit (*R*^2^) of 0.9703, 0.9938, and 0.9872 for fungal biomass, DON, and ZEA, respectively (Supplementary Table S1). The regression models have confirmed that effect of SaZnO NPs on fungal growth and mycotoxins production was effective and dose-dependent.

### Antifungal Mechanism of SaZnO NPs

#### Analysis of ROS Generation and Lipid Peroxidation

**FIGURE 4:**
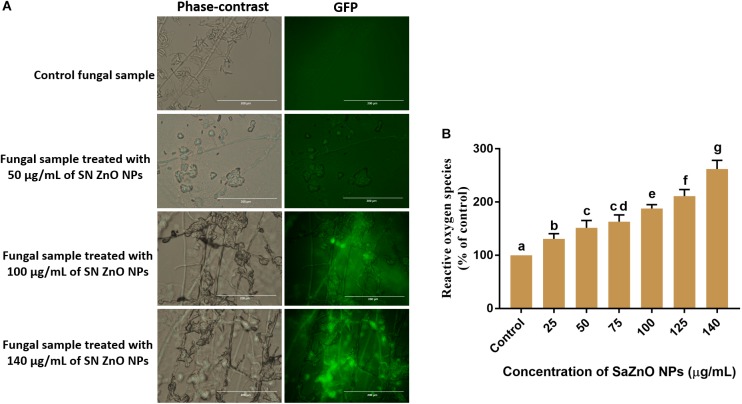
Assessment of different concentration of SaZnO NPs on generation of intracellular reactive oxygen species (ROS). **(A)** phase-contrast and green fluorescent protein (GFP) images of control and SaZnO NPs treated fungal samples. **(B)** Dose-dependent effect of SaZnO NPs on accumulation of ROS in fungi. The data was analyzed by one-way ANOVA according to Tukey’s multiple comparison test and experimental clusters denoted with different alphabetic letters were significant (*p* < 0.05).

**FIGURE 5:**
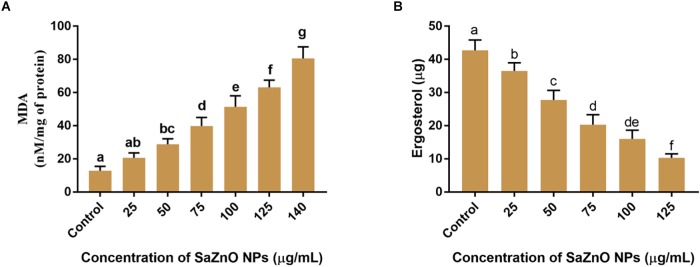
**(A)** Effect of different concentration of SaZnO NPs on fungal lipid peroxidation (MDA release). **(B)** Effect of different concentration of SaZnO NPs on fungal ergosterol content. The data was analyzed by one-way ANOVA according to Tukey’s multiple comparison test and experimental clusters denoted with different alphabetic letters were significant (*p* < 0.05).

The effect of SaZnO NPs on ROS generation and lipid peroxidation was determined by DCFH-DA staining and MDA analysis, respectively. The phase-contrast and GFP images of control and SaZnO NPs treated fungal samples of ROS investigation were depicted in Figure 4A. The accumulation of ROS molecules was significantly (*P* < 0.05) high in SaZnO NPs treated samples compared to control (Figure 4B). The results determined that accumulation of ROS molecules was directly proportional to the dose of SaZnO NPs and it shows the dose-dependent manner. Likewise, lipid peroxidation was also highly influenced by SaZnO NPs and MDA levels were increased in fungi with the treatment of SaZnO NPs (Figure 5A). Furthermore, the accumulation of MDA levels was directly proportional to SaZnO NPs and it was dose-dependent and in accordance with the results of ROS analysis.

#### Analysis of Ergosterol Content and Membrane Integrity

Ergosterol is a key sterol component in the cell membrane of fungi and helpful for guarding the permeability and fluidity of membrane through interactions with phospholipids and other components of the membrane. The ergot is present only in fungi and yeast, and it is absent in animals. Therefore, disruption of ergot biosynthesis is considered as one of the particulars focuses for the advancement of new antifungal agents (Ahmad et al., 2010). The effect of SaZnO NPs on the ergosterol biosynthesis of *F. graminearum* was depicted in Figure 5B. A quantity of 42.7 ± 3.10 μg of ergosterol was measured in SaZnO NPs untreated control sample. Whereas, biosynthesis of ergosterol was reduced with treatment of SaZnO NPs and showed the dose-dependent mode of decrease. The fungal growth was absent at 140 μg/mL of SaZnO NPs and ergosterol was not determined.

**FIGURE 6:**
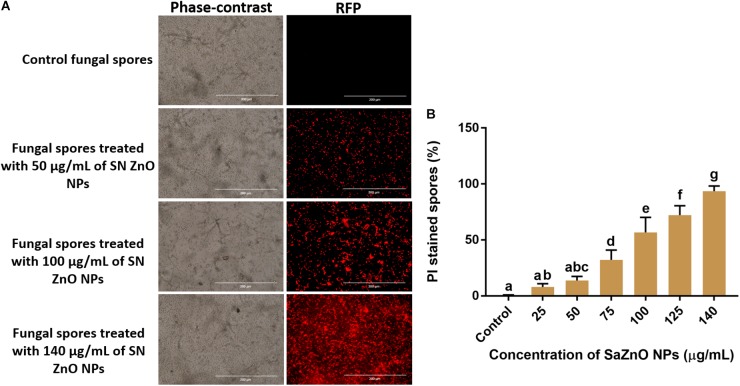
Assessment of different concentration of SaZnO NPs on membrane integrity of fungal spores. **(A)** phase-contrast and red fluorescent protein (RFP) images of control and SaZnO NPs treated fungal spore samples. **(B)** Flowcytometric estimation of percentage of propidium iodide (PI) stained fungal spores. The data was analyzed by one-way ANOVA according to Tukey’s multiple comparison test and experimental clusters denoted with different alphabetic letters were significant (*p* < 0.05).

Sustaining the membrane integrity is most necessary for viable cells and therefore, is often used as an index of living cells. The loss of membrane structure and integrity release cytochrome c and activate the death of cells by the apoptosis process. The membrane integrity of fungal spores is generally estimated by a fluorometric method using PI staining. The PI is a fluorescent stain that intensely binds to DNA and is incapable to enter the membrane of viable cells, and however, it uniquely stains DNA of non-viable cells by crossing the damaged cell membrane. Phase-contrast images and its corresponding RFP images of control and SaZnO NPs treated fungal spores were depicted in Figure 6A. The fluorescence intensity of fungal spores was enhanced on the treatment of SaZnO NPs related to untreated control. The percentage PI stained spores were recorded by flow cytometry and their number was increased with the dose of SaZnO NPs (Figure 6B). The results evidently proved that SaZnO NPs induces the detriments in membrane integrity of fungal spores and thereby promotes the death of fungi in a dose-dependent fashion.

#### Assessment of Micromorphology of Macroconidia

**FIGURE 7:**
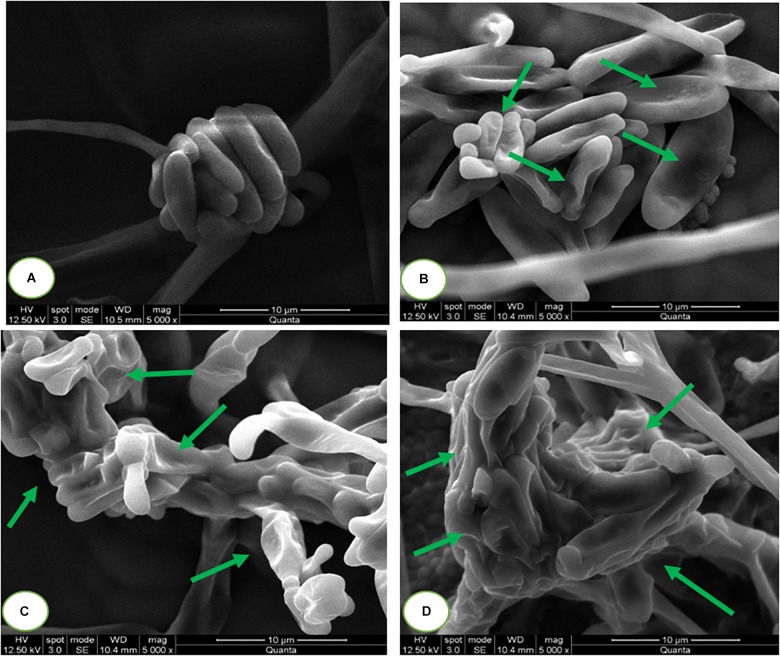
Scanning electron microscopic images of fungal macroconidia at **(A)** 0 (Control), **(B)** 100, **(C)** 125, and **(D)** 140 μg/mL of SaZnO NPs.

The micromorphology of fungal macroconidia was observed using SEM under environmental mode (Figure 7). The macroconidia in the control sample have exhibited healthy morphological characters, such as smooth, turgid, and regular. Whereas, SaZnO NPs treated macroconidia have shown detrimental micromorphological features, including irregular, wrinkled, disrupted, shrunk, and blebs. Captivatingly, severe detrimental micro-morphological changes in fungal macroconidia were noticed at the high dose of SaZnO NPs related lower doses and control (Figure 7A–D). The study decided that fungicidal action of SaZnO NPs is due to detrimental damage of macroconidia, and it could be due to the surge of intracellular ROS and lipid peroxidation, and the collapse of ergosterol content and membrane integrity.

## Discussion

Since last decade, nanotechnology has offered various beneficial assistances to the agriculture and food industry. Which include antimicrobial agents, nanobiosensors, food packaging material, catalysts, etc. (Zhu and Deng, 2017; Zhu H. et al., 2017; Zhu L. et al., 2017; Siddaiah et al., 2018; Kalagatur et al., 2018a; Gunti et al., 2019). As of now, the contagious pervasion and mycotoxin pollution of nourishment is one of the principals stresses of agribusiness and sustenance industry (Adeyeye, 2016). Right now, microbiologists have given extraordinary significance to seek novel antifungal and antimycotoxin mixes, which ought to be unique in relation to as of now accessible antifungal and antimycotoxin substances or ought to have productive antifungal and antimycotoxin movement. Be that as it may, best of our insight utilization of nanomaterials for controlling development and creation of mycotoxins in rare. In this way, an exertion made to investigate the biosynthesized SaZnO NPs for controlling of development and mycotoxins of wrecking plant pathogen *F. graminearum*.

The antifungal mechanism of SaZnO NPs on *F. graminearum* was unveiled by evaluating the aggregation of ROS, lipid peroxidation, ergosterol content, membrane integrity, and micromorphology of macroconidia. The antimicrobial activity of ZnO NPs directly correlated to detrimental action of radicals such as ^●^OH, H^+^, HO_2_^●^ and H_2_O_2_ on cell wall and other cellular constituents. The ZnO NPs with deformities can be enacted by both UV and noticeable light electron-gap sets (*e^-^h^+^*). Initially, protons and electrons generate from SaZnO NPs by action of light energy. Following, protons react with water molecules and form ^●^OH and electrons react with O_2_ and generate ^●^O_2_. Next, combination of ^●^OH and ^●^O_2_ generates HO_2_^●^ and succeeding, HO_2_^●^ recombines with proton and electron and generate H_2_O_2_ (Zhang et al., 2007; Padmavathy and Vijayaraghavan, 2008). The overall generation of radicals is represented in below reaction.

$$ZnO+light\to proton(h^{+})+electron(e^{-})$$

$$h^{+}+H_{2}O\to OH^{•}+H^{+}:e^{+}+O_{2}\to^{•}O_{2}$$

$$OH^{•}+{\;}^{•}O_{2}\to HO_{2}{}^{•}$$

$$HO_{2}{}^{•}+H^{+}+e^{-}\to H_{2}O_{2}$$

The microorganisms on their cell wall bear a negative charge while SaZnO NPs carry a positive charge along these lines this cooperation makes an electromagnetic fascination between the organisms and nanoparticles. As SaZnO NPs on coming into contact with the surface of microbes produce ROS by action of light. Antifungal mechanisms of SaZnO NPs might be credited to the ROS such as hydroxyl radicals (OH^●^) superoxides (^●^O_2_) and hydrogen peroxide (H_2_O_2_) (Prasanna and Vijayaraghavan, 2017; Wang et al., 2017; Youssef et al., 2017).

In a biological context, intracellular ROS assume an unequivocal part as couriers in quality articulation and cell flagging and expressly starts oxidative pressure intervened apoptosis (Simon et al., 2000). The ROS molecules are formed as byproducts in mitochondrial electron transport by various inward and outer pressure factors, for example, radiations, warm treatment, supplement lack, drugs, anti-infection agents, antimicrobial peptides, nanoparticles, saltiness, microbial poisons, metals, and so on (Matai et al., 2014; Zhang Z.-Z. et al., 2017; Kalagatur et al., 2018d,e). The elevated ROS molecules attack the lipid molecules comprising of carbon-carbon double bonds in polyunsaturated fatty acids and fetch lipid peroxidation (Sebaaly et al., 2016). Furthermore, elevation of ROS levels eventually impairments the proteins, lipids, and nucleic acids, and triggers oxidative-stress mediated apoptosis by releasing mitochondrial cytochrome c and death receptors.

Antifungal and antimycotoxin activities of SaZnO NPs might be due to an elevation of ROS and thus, it could induce death of fungi by oxidative-stress mediated apoptosis. In support of the present study, several studies have evidenced that nanoparticles (Mitra et al., 2017), physical agents (Calado et al., 2014; Kalagatur et al., 2018d,e), synthetic fungicides (Delattin et al., 2014) and bio-fungicides (Sebaaly et al., 2016; Sellamani et al., 2016) could induce the death of fungi through oxidative-stress mediated apoptosis by elevation of ROS. Specifically concerning to nanomaterials, few reports were available on antifungal and antimycotoxin capabilities of nanomaterials. Best of our knowledge, until the date, no report is available on antifungal and antimycotoxin abilities of biofabricated ZnO NPs on *F. graminearum* and present study is first report. Most recently, Hernández-Meléndez et al. (2018) reported that flower-shaped ZnO inhibit the growth and aflatoxin production of *A. flavus* by multiple degenerative alterations in fungi via ROS generation. Besides, Savi et al. (2013) proved that zinc compounds could generate intracellular ROS and exhibit strong inhibitory activity on growth and fumonisin mycotoxin production of *F. verticillioides* by detrimental action on hyphae and conidia. Captivatingly, Mitra et al. (2017) reported that citrate decorated silver nanoparticles independently inhibit the fungal growth and aflatoxin production in *A. parasiticus* and revealed that silver nanoparticles inhibit aflatoxin production by downregulating the aflatoxin biosynthesis genes at its below the lethal dose.

In addition, our study revealed that SaZnO NPs might exhibit potent fungicidal activity on *F. graminearum* through inhibition of ergosterol biosynthesis and disrupting the membrane integrity. In support of the present result, Sellamani et al. (2016) demonstrated that *Pediococcus pentosaceus* isolated from dairy products has exhibited potent antifungal activity on *F. graminearum* by inhibiting the ergosterol biosynthesis. Ahmad et al. (2010) have proven that the antifungal activity of thymol and carvacrol is due to disruption of ergosterol biosynthesis and membrane integrity. In the same way, Prasher et al. (2018) have confirmed that green synthesized silver nanoparticles exhibit the potent antifungal activity through disturbing ergosterol biosynthesis and membrane integrity. However, molecular mechanism involved in inhibition of growth and mycotoxins production of fungi by nanomaterials is unclear and exhaustive studies are needed.

## Conclusion

In conclusion, we have synthesized a highly facile, non-toxic and inexpensive approach to the green synthesis of SaZnO NPs. The SaZnO NPs has presented potent inhibitory activity against growth and mycotoxin production of *F. graminearum*. Furthermore, the antifungal mechanism of SaZnO NPs on *F. graminearum* was established by assessing the membrane integrity, ROS generation, lipid peroxidation, ergosterol content, and micromorphology of macroconidia. These studies demonstrated that SaZnO NPs has upraised the ROS levels and lipid peroxidation, and depleted the ergosterol content, and detrimentally altered the membrane integrity and micromorphology of macroconidia. The proposed antifungal mode suggested that SaZnO NPs could efficiently restrain the growth and mycotoxin production of devastating plant pathogen *F. graminearum* and could be used in novel fungicide formulations as the potent substitute of synthetic fungicides for agriculture and food industry.

## Author Contributions

TL and CS designed the experiments. TL and NK performed the experiments. TL, CS, CM, VG, and NK wrote the manuscript. TL, CM, CS, SN, VM, BA, AH, EA_A, AA, and BP reviewed and finalized the manuscript. All authors read and approved the final version of this manuscript.

## Conflict of Interest Statement

The authors declare that the research was conducted in the absence of any commercial or financial relationships that could be construed as a potential conflict of interest.

## Supplementary Material

FIGURE S1 Linear regression curves for inhibitory effect of SaZnO NPs on **(A)** mycelial biomass, **(B)** deoxynivalenol (DON), and **(C)** zearalenone (ZEA) of *F. graminearum*.

TABLE S1 Linear regression curve fit for dose-dependent inhibitory effect of SaZnO NPs on fungal growth (mycelial biomass), deoxynivalenol (DON), and zearalenone (ZEA) of *F. graminearum* in broth culture.

## Abbreviations

ANOVA: analysis of variance
CZA: czapek dox agar
CZB: czapek dox broth
DCFH-DA: dichloro-dihydro-fluorescein diacetate
DON: deoxynivalenol
DPBS: dulbecco’s phosphate-buffered saline
EDX: energy dispersive X-ray diffractive
FAO: Food and Agriculture Organization
FHB: fusarium head blight
FTIR: fourier-transform infrared spectroscopy
GC-MS: gas chromatography-mass spectroscopy
GFP: green fluorescent protein
HPLC: high-performance liquid chromatography
MDA: malondialdehyde
NIV: nivalenol
PI: propidium iodide
ROS: reactive oxygen species
SaZnO NPs: *Syzygium aromaticum* zinc oxide nanoparticles
SEM: scanning electron microscope
TEM: transmission electron microscopy
UV: ultraviolet
XRD: X-ray diffraction
ZEA: zearalenone
ZnO: zinc oxide

## References

[B1] Adeyeye S. A. O. (2016). Fungal mycotoxins in foods: a review. *Cogent. Food Agric.* 2:1213127 10.1080/23311932.2016.1213127

[B2] Ahmad A. Khan A. Manzoor N. Khan L. A. (2010). Evolution of ergosterol biosynthesis inhibitors as fungicidal against Candida. *Microb. Pathog.* 48 35–41. 10.1016/j.micpath.2009.10.001 19835945

[B3] Ahmed S. Annu Chaudhry S. A. Ikram S. (2017). A review on biogenic synthesis of ZnO nanoparticles using plant extracts and microbes: a prospect towards green chemistry. *J. Photochem. Photobiol. B.* 166 272–284. 10.1016/j.jphotobiol.2016.12.011 28013182

[B4] Alexandratos N. Bruinsma J. (2012). *World Agriculture Towards 2030/2050: the 2012 Revision.* Rome: FAO.

[B5] Ali S. Prasad R. Mahmood A. Routray I. Shinkafi T. S. Sahin K. (2014). Eugenol-rich fraction of syzygium aromaticum (Clove) reverses biochemical and histopathological changes in liver cirrhosis and inhibits hepatic cell proliferation. *J. Cancer Prev.* 19 288–300. 10.15430/JCP.2014.19.4.288 25574464 PMC4285960

[B6] Aulakh J. Regmi A. (2013). *Post-Harvest Food Losses Estimation-Development of Consistent Methodology.* Rome: FAO.

[B7] Basnet P. Inakhunbi Chanu T. Samanta D. Chatterjee S. (2018). A review on bio-synthesized zinc oxide nanoparticles using plant extracts as reductants and stabilizing agents. *J. Photochem. Photobiol. B.* 183 201–221. 10.1016/j.jphotobiol.2018.04.036 29727834

[B8] Bhuiyan M. N. I. (2012). Constituents of the essential oil from leaves and buds of clove (*Syzigium caryophyllatum* (L.) Alston). *Afr. J. Pharm. Pharmaco.* 6 1260–1263. 10.5897/AJPP10.004

[B9] Calado T. Venâncio A. Abrunhosa L. (2014). Irradiation for mold and mycotoxin control: a review. *Compr. Rev. Food Sci. Food Saf.* 13 1049–1061. 10.1111/1541-4337.12095

[B10] Cansian R. L. Vanin A. B. Orlando T. Piazza S. P. Puton B. M. S. Cardoso R. I. (2016). Toxicity of clove essential oil and its ester eugenyl acetate against *Artemia salina*. *Braz. J. Biol.* 77 155–161. 10.1590/1519-6984.12215 27382998

[B11] Chatterjee D. Bhattacharjee P. (2015). Use of eugenol-lean clove extract as a flavoring agent and natural antioxidant in mayonnaise: product characterization and storage study. *J. Food Sci. Technol.* 52 4945–4954. 10.1007/s13197-014-1573-6 26243914 PMC4519447

[B12] Chen H. Diao J. Li Y. Chen Q. Kong B. (2016). The effectiveness of clove extracts in the inhibition of hydroxyl radical oxidation-induced structural and rheological changes in porcine myofibrillar protein. *Meat Sci.* 111 60–66. 10.1016/j.meatsci.2015.08.017 26340742

[B13] Chen X. Ren L. Li M. Qian J. Fan J. Du B. (2017a). Effects of clove essential oil and eugenol on quality and browning control of fresh-cut lettuce. *Food Chem.* 214 432–439. 10.1016/j.foodchem.2016.07.101 27507495

[B14] Chen X. Wu Z. Liu D. Gao Z. (2017b). Preparation of ZnO photocatalyst for the efficient and rapid photocatalytic degradation of azo dyes. *Nanoscale Res. Lett.* 12 143–153. 10.1186/s11671-017-1904-4 28235375 PMC5319938

[B15] Delattin N. Cammue B. P. Thevissen K. (2014). Reactive oxygen species-inducing antifungal agents and their activity against fungal biofilms. *Future Med. Chem.* 6 77–90. 10.4155/fmc.13.189 24358949

[B16] Escrivá L. Font G. Manyes L. (2015). In vivo toxicity studies of fusarium mycotoxins in the last decade: a review. *Food Chem. Toxicol.* 78 185–206. 10.1016/j.fct.2015.02.005 25680507

[B17] FAO (2012). *The State of Food Insecurity in the World 2012. Economic Growth is Necessary but not Sufficient to Accelerate Reduction of Hunger and Malnutrition.* Rome: FAO. 10.3945/an.112.003343 PMC3648734 23319131

[B18] George E. Kasipandi M. Vekataramana M. Kumar K. N. Allen J. A. Parimelazhagan T. (2016). In vitro anti-oxidant and cytotoxic analysis of Pogostemon mollis Benth. *Bangladesh J. Pharmacol.* 11 148–158. 10.3329/bjp.v11i1.24157

[B19] Gunti L. Dass R. S. Kalagatur N. K. (2019). Phytofabrication of Selenium Nanoparticles from Emblica officinalis fruit extract and exploring its biopotential applications: antioxidant. Antimicrobial, and Biocompatibility. *Front. Microbiol.* 10:931. 10.3389/fmicb.2019.00931 31114564 PMC6503097

[B20] Gustavsson J. Cederberg C. Sonesson U. van Otterdijk R. Meybeck A. (2011). *Global Food Losses and Food Waste.* Rome: FAO.

[B21] Hamed S. F. Sadek Z. Edris A. (2012). Antioxidant and antimicrobial activities of clove cud essential oil and eugenol nanoparticles in alcohol-free microemulsion. *J. Oleo Sci.* 61 641–648. 10.5650/jos.61.641 23138253

[B22] Hernández-Meléndez D. Salas-Téllez E. Zavala-Franco A. Téllez G. Méndez-Albores A. Vázquez-Durán A. (2018). Inhibitory effect of flower-shaped zinc oxide nanostructures on the growth and aflatoxin production of a highly toxigenic strain of aspergillus flavus link. *Materials* 11:1265. 10.3390/ma11081265 30042297 PMC6117727

[B23] Iorgu A. I. Berger D. Alexandrescu L. Vasile B. S. Matei C. (2013). Synthesis of photoluminescent pure and doped cadmium sulfide by reverse microemulsion method. *Chalcogenide Lett.* 10 525–531.

[B24] Kadiyala U. Turali-Emre E. S. Bahng J. H. Kotov N. A. VanEpps J. S. (2018). Unexpected insights into antibacterial activity of zinc oxide nanoparticles against methicillin resistant *Staphylococcus aureus* (MRSA). *Nanoscale* 10 4927–4939. 10.1039/C7NR08499D 29480295 PMC5847298

[B25] Kalagatur N. K. Ghosh O. S. N. Sundararaj N. Mudili V. (2018a). Antifungal activity of chitosan nanoparticles encapsulated with *Cymbopogon martinii* essential oil on plant pathogenic fungi Fusarium graminearum. *Front. Pharmacol.* 9:610. 10.3389/fphar.2018.00610 29928233 PMC5997812

[B26] Kalagatur N. K. Kamasani J. R. Mudili V. (2018b). Assessment of detoxification efficacy of irradiation on zearalenone mycotoxin in various fruit juices by response surface methodology and elucidation of its in-vitro toxicity. *Front. Microbiol.* 9:2937. 10.3389/fmicb.2018.02937 30555450 PMC6284055

[B27] Kalagatur N. K. Kamasani J. R. Mudili V. Krishna K. Chauhan O. P. Sreepathi M. H. (2018c). Effect of high pressure processing on growth and mycotoxin production of Fusarium graminearum in maize. *Food Biosci.* 21 53–59. 10.1016/j.fbio.2017.11.005

[B28] Kalagatur N. K. Kamasani J. R. Siddaiah C. Gupta V. K. Krishna K. Mudili V. (2018d). Combinational inhibitory action of *Hedychium spicatum* L. essential oil and γ-radiation on growth rate and mycotoxins content of *Fusarium graminearum* in maize: response surface methodology. *Front. Microbiol.* 9:1511. 10.3389/fmicb.2018.01511 30108550 PMC6079234

[B29] Kalagatur N. K. Mudili V. Kamasani J. R. Siddaiah C. (2018e). Discrete and combined effects of Ylang-Ylang (*Cananga odorata*) essential oil and gamma irradiation on growth and mycotoxins production by *Fusarium graminearum* in maize. *Food Control* 94 276–283. 10.1016/j.foodcont.2018.07.030

[B30] Kalagatur N. K. Karthick K. Allen J. A. Ghosh N. Sivaraman O. Chandranayaka S. (2017). Application of activated carbon derived from seed shells of *Jatropha curcas* for decontamination of zearalenone mycotoxin. *Front. Pharmacol.* 8:760. 10.3389/fphar.2017.00760 29114225 PMC5660729

[B31] Kalagatur N. K. Mudili V. Siddaiah C. Gupta V. K. Natarajan G. Sreepathi M. H. (2015). Antagonistic activity of Ocimum sanctum L. essential oil on growth and zearalenone production by Fusarium graminearum in maize grains. *Front. Microbiol.* 6:892. 10.3389/fmicb.2015.00892 26388846 PMC4558530

[B32] Kavyashree D. Shilpa C. J. Nagabhushana H. Daruka Prasad B. Sreelatha G. L. Sharma S. C. (2015). ZnO superstructures as an antifungal for effective control of malassezia furfur, dermatologically prevalent yeast: prepared by Aloe vera assisted combustion method. *ACS Sustain. Chem. Eng.* 3 1066–1080. 10.1021/sc500784p

[B33] Kheawfu K. Pikulkaew S. Hamamoto H. Sekimizu K. Okonogi S. (2017). Influence of clove oil and eugenol on muscle contraction of silkworm (*Bombyx mori*). *Drug Discov. Ther.* 11 64–69. 10.5582/ddt.2017.01012 28458297

[B34] Kovács J. K. Felsõ P. Makszin L. Pápai Z. Horváth G. Ábrahám H. (2016). Antimicrobial and virulence-modulating effects of clove essential oil on the foodborne pathogen *Campylobacter jejuni*. *Appl. Environ. Microbiol.* 82 6158–6166. 10.1128/AEM.01221-16 27520816 PMC5068167

[B35] Kumar K. N. Venkataramana M. Allen J. A. Chandranayaka S. Murali H. S. Batra H. V. (2016). Role of *Curcuma longa* L. essential oil in controlling the growth and zearalenone production of *Fusarium graminearum*. *LWT-Food Sci. Technol.* 69 522–528. 10.1016/j.lwt.2016.02.005

[B36] Lakshmeesha T. R. Sateesh M. K. Prasad B. D. Sharma S. C. Kavyashree D. Chandrasekhar M. (2014). Reactivity of crystalline ZnO superstructures against fungi and bacterial pathogens: synthesized using Nerium oleander leaf extract. *Cryst. Growth Des.* 14 4068–4079. 10.1021/cg500699z

[B37] Matai I. Sachdev A. Dubey P. Uday Kumar S. Bhushan B. Gopinath P. (2014). Antibacterial activity and mechanism of Ag-ZnO nanocomposite on S. aureus and GFP-expressing antibiotic resistant E. coli. *Colloids Surf. B Biointer.* 115 359–367. 10.1016/j.colsurfb.2013.12.005 24412348

[B38] Mitra C. Gummadidala P. M. Afshinnia K. Merrifield R. C. Baalousha M. Lead J. R. (2017). Citrate-coated silver nanoparticles growth-independently inhibit aflatoxin synthesis in *Aspergillus parasiticus*. *Environ. Sci. Technol.* 51 8085–8093. 10.1021/acs.est.7b01230 28618218

[B39] Mudili V. Siddaih C. N. Nagesh M. Garapati P. Kumar N. K. Murali H. S. (2014). Mould incidence and mycotoxin contamination in freshly harvested maize kernels originated from India. *J. Sci. Food Agric.* 94 2674–2683. 10.1002/jsfa.6608 24609945

[B40] Padmavathy N. Vijayaraghavan R. (2008). Enhanced bioactivity of ZnO nanoparticles-an antimicrobial study. *Sci. Technol. Adv. Mater.* 9 35004–35011. 10.1088/1468-6996/9/3/035004 27878001 PMC5099658

[B41] Pasquali M. Beyer M. Logrieco A. Audenaert K. Balmas V. Basler R. (2016). A European database of *Fusarium graminearum* and *F. culmorum* trichothecene genotypes. *Front. Microbiol.* 7:406. 10.3389/fmicb.2016.00406 27092107 PMC4821861

[B42] Prasanna L. V. Vijayaraghavan R. (2017). Chemical manipulation of oxygen vacancy and antibacterial activity in ZnO. *Mater. Sci. Eng. C* 77 1027–1034. 10.1016/j.msec.2017.03.280 28531975

[B43] Prashanth G. K. Prashanth P. A. Nagabhushana B. M. Ananda S. Krishnaiah G. M. Nagendra H. G. (2018). Comparison of anticancer activity of biocompatible ZnO nanoparticles prepared by solution combustion synthesis using aqueous leaf extracts of *Abutilon indicum*. Melia azedarach and Indigofera tinctoria as biofuels. *Artif. Cells Nanomed. Biotechnol.* 46 968–979. 10.1080/21691401.2017.1351982 28719999

[B44] Prasher P. Singh M. Mudila H. (2018). Green synthesis of silver nanoparticles and their antifungal properties. *Bionanoscience* 8 254–263. 10.1007/s12668-017-0481-4

[B45] Reddy A. J. Kokila M. K. Nagabhushana H. Rao J. L. Shivakumara C. Nagabhushana B. M. (2011). Combustion synthesis, characterization and raman studies of ZnO nanopowders. *Spectrochim. Acta A Mol. Biomol. Spectrosc.* 81 53–58. 10.1016/j.saa.2011.05.043 21764361

[B46] Richard J. L. (2007). Some major mycotoxins and their mycotoxicoses-An overview. *Int. J. Food Microbiol.* 119 3–10. 10.1016/j.ijfoodmicro.2007.07.019 17719115

[B47] Savi G. D. Vitorino V. Bortoluzzi A. J. Scussel V. M. (2013). Effect of zinc compounds on Fusarium verticillioides growth, hyphae alterations, conidia, and fumonisin production. *J. Sci. Food Agric.* 93 3395–3402. 10.1002/jsfa.6271 23775536

[B48] Sebaaly C. Charcosset C. Stainmesse S. Fessi H. Greige-Gerges H. (2016). Clove essential oil-in-cyclodextrin-in-liposomes in the aqueous and lyophilized states: from laboratory to large scale using a membrane contactor. *Carbohyd. Polym.* 138 75–85. 10.1016/j.carbpol.2015.11.053 26794740

[B49] Sellamani M. Kalagatur N. K. Siddaiah C. Mudili V. Krishna K. Natarajan G. (2016). Antifungal and zearalenone inhibitory activity of *Pediococcus pentosaceus* isolated from dairy products on Fusarium graminearum. *Front. Microbiol.* 7:890. 10.3389/fmicb.2016.00890 27379035 PMC4904835

[B50] Sheweita S. A. El-Hosseiny L. S. Nashashibi M. A. (2016). Protective effects of essential oils as natural antioxidants against hepatotoxicity induced by cyclophosphamide in mice. *PLoS One* 11:e0165667. 10.1371/journal.pone.0165667 27802299 PMC5089748

[B51] Siddaiah C. N. Prasanth K. V. H. Satyanarayana N. R. Mudili V. Gupta V. K. Kalagatur N. K. (2018). Chitosan nanoparticles having higher degree of acetylation induce resistance against pearl millet downy mildew through nitric oxide generation. *Sci. Rep.* 8:2485. 10.1038/s41598-017-19016-z 29410438 PMC5802724

[B52] Simon H. U. Haj-Yehia A. Levi-Schaffer F. (2000). Role of reactive oxygen species (ROS) in apoptosis induction. *Apoptosis* 5 415–418. 10.1023/A:1009616228304 11256882

[B53] Smith M. C. Madec S. Coton E. Hymery N. (2016). Natural co-occurrence of mycotoxins in foods and feeds their in vitro combined toxicological effects. *Toxins* 8:94. 10.3390/toxins8040094 27023609 PMC4848621

[B54] Suresh D. Shobharani R. M. Nethravathi P. C. Pavan Kumar M. A. Nagabhushana H. Sharma S. C. (2015). Artocarpus gomezianus aided green synthesis of ZnO nanoparticles: luminescence, photocatalytic and antioxidant properties. *Spectrochim. Acta A Mol. Biomol. Spectrosc.* 141 128–134. 10.1016/j.saa.2015.01.048 25668693

[B55] Velmurugan P. Park J. H. Lee S. M. Yi Y. J. Cho M. Jang J. S. (2016). Eco-friendly approach towards green synthesis of zinc oxide nanocrystals and its potential applications. *Artif. Cells Nanomed. Biotechnol.* 44 1537–1543. 10.3109/21691401.2015.1059840 26135054

[B56] Venkataramana M. Nayaka S. C. Anand T. Rajesh R. Aiyaz M. Divakara S. T. (2014). Zearalenone induced toxicity in SHSY-5Y cells: the role of oxidative stress evidenced by N-acetyl cysteine. *Food Chem. Toxicol.* 65 335–342. 10.1016/j.fct.2013.12.042 24412706

[B57] Vijayakumar S. Malaikozhundan B. Shanthi S. Vaseeharan B. Thajuddin N. (2017). Control of biofilm forming clinically important bacteria by green synthesized ZnO nanoparticles and its ecotoxicity on Ceriodaphnia cornuta. *Microb. Pathog.* 107 88–97. 10.1016/j.micpath.2017.03.019 28330748

[B58] Wang Z. Zhang L. Liu Z. Sang L. Yang L. Chen Q. (2017). The antibacterial polyamide 6-ZnO hierarchical nanofibers fabricated by atomic layer deposition and hydrothermal growth. *Nanoscale Res. Lett.* 12 421–429. 10.1186/s11671-017-2162-1 28637349 PMC5478553

[B59] Youssef A. M. El-Nahrawy A. M. Abou Hammad A. B. (2017). Sol-gel synthesis and characterizations of hybrid chitosan-PEG/calcium silicate nanocomposite modified with ZnO-NPs and (E102) for optical and antibacterial applications. *Int. J. Biol. Macromol.* 97 561–567. 10.1016/j.ijbiomac.2017.01.059 28108409

[B60] Zhang P. Zhang W. Wang J. Jiang K. Zhang J. Li W. (2017). The electro-optic mechanism and infrared switching dynamic of the hybrid multilayer VO_2_/Al:ZnO heterojunctions. *Sci. Rep.* 7 4425–4439. 10.1038/s41598-017-04660-2 28667297 PMC5493620

[B61] Zhang Z.-Z. Xu J.-J. Shi Z.-J. Cheng Y.-F. Ji Z.-Q. Deng R. (2017). Short-term impacts of Cu, CuO, ZnO and Ag nanoparticles (NPs) on anammox sludge CuNPs make a difference. *Bioresour. Technol.* 235 281–291. 10.1016/j.biortech.2017.03.135 28371766

[B62] Zhang Y. G. Ma L. L. Li J. L. Yu Y. (2007). In situ Fenton reagent generated from TiO2/Cu2O composite film: a new way to utilize TiO2 under visible light irradiation. *Environ. Sci. Technol.* 41 6264–6269. 10.1021/es070345i 17937313

[B63] Zheng L. Wan Y. Qi P. Sun Y. Zhang D. Yu L. (2017). Lectin functionalized ZnO nanoarrays as a 3D nano-biointerface for bacterial detection. *Talanta* 167 600–606. 10.1016/j.talanta.2017.03.007 28340767

[B64] Zhu H. Xu X. Tian X. Tang J. Liang H. Chen L. (2017). A thresholdless tunable raman nanolaser using a ZnO-graphene superlattice. *Adv. Mater.* 29:1604351. 10.1002/adma.201604351 27862431

[B65] Zhu L. Wang L. Xue F. Chen L. Fu J. Feng X. (2017). Piezo-phototronic effect enhanced flexible solar cells based on n-ZnO/p-SnS core–shell nanowire array. *Adv. Sci.* 4 1600185–1600192. 10.1002/advs.201600185 28105394 PMC5238743

[B66] Zhu J. Deng D. (2017). Ammonia-assisted wet-chemical synthesis of ZnO microrod arrays on substrates for microdroplet transfer. *Langmuir* 33 6143–6150. 10.1021/acs.langmuir.7b00921 28603993

