# Supplementary figures and images for: Biofabrication of Zinc Oxide Nanoparticles With Syzygium aromaticum Flower Buds Extract and Finding Its Novel Application in Controlling the Growth and Mycotoxins of Fusarium graminearum
Source: Front Microbiol. 2019 Jun 12;10:1244. doi: 10.3389/fmicb.2019.01244 (PMC6582371; doi:10.3389/fmicb.2019.01244)

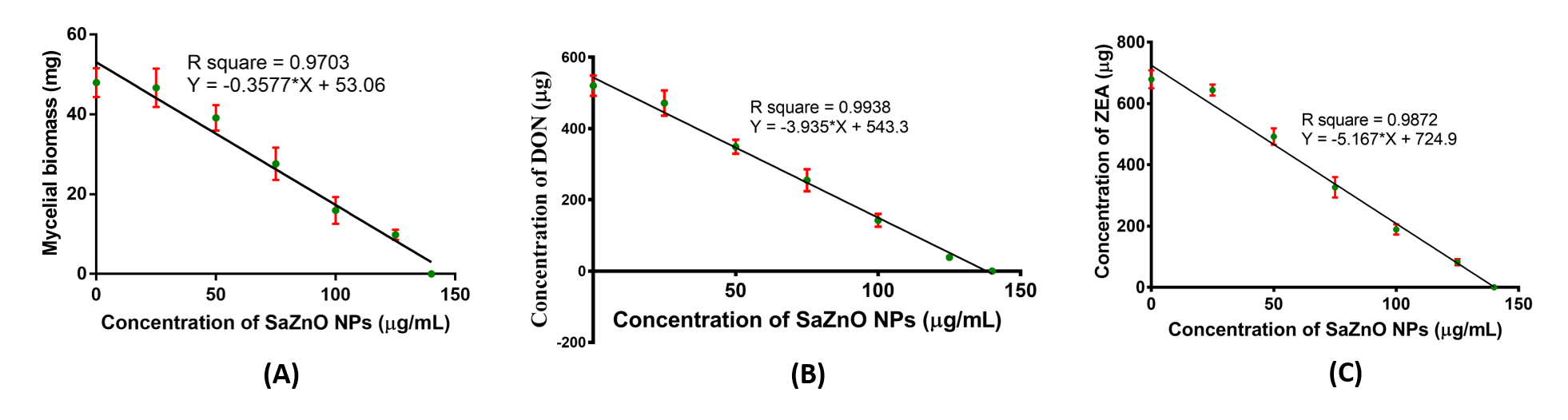

Supplement: FIGURE S1 — Linear regression curves for inhibitory effect of SaZnO NPs on (A) mycelial biomass, (B) deoxynivalenol (DON), and (C) zearalenone (ZEA) of F. graminearum. [file Image_1.TIF]
