# Supplementary material for: Biofabrication of Zinc Oxide Nanoparticles With Syzygium aromaticum Flower Buds Extract and Finding Its Novel Application in Controlling the Growth and Mycotoxins of Fusarium graminearum
Source: Front Microbiol. 2019 Jun 12;10:1244. doi: 10.3389/fmicb.2019.01244 (PMC6582371; doi:10.3389/fmicb.2019.01244)
Supplement: TABLE S1 — Linear regression curve fit for dose-dependent inhibitory effect of SaZnO NPs on fungal growth (mycelial biomass), deoxynivalenol (DON), and zearalenone (ZEA) of F. graminearum in broth culture. [file Table_1.DOCX]

**Supplementary Table 1.** Linear regression curve fit for dose-dependent inhibitory effect of SaZnO NPs on fungal growth (mycelial biomass), deoxynivalenol (DON), and zearalenone (ZEA) of *F. graminearum* in broth culture.

|  | Mycelial biomass (mg) | DON (µg) | ZEA (µg) |
| --- | --- | --- | --- |
| Best-fit values ± SE |  |  |  |
| Slope | -0.3577 ± 0.02801 | -3.935 ± 0.1388 | -5.167 ± 0.2633 |
| Y-intercept | 53.06 ± 2.459 | 543.3 ± 12.19 | 724.9 ± 23.12 |
| X-intercept | 148.3 | 138.1 | 140.3 |
| 1/slope | -2.796 | -0.2541 | -0.1935 |
| 95% confidence intervals |  |  |  |
| Slope | - 0.4297 to -0.2857 | -4.292 to -3.579 | -5.844 to -4.49 |
| Y-intercept | 46.74 to 59.38 | 512 to 574.6 | 665.4 to 784.3 |
| X-intercept | 133 to 170 | 131.3 to 145.9 | 130.5 to 152.4 |
| Goodness of fit |  |  |  |
| R square | 0.9703 | 0.9938 | 0.9872 |
| Sy.x | 3.552 | 17.6 | 33.39 |
| Is slope significantly non-zero? |  |  |  |
| F | 163.1 | 804.2 | 385.2 |
| DFn, DFd | 1, 5 | 1, 5 | 1, 5 |
| *P* value | < 0.005 | < 0.005 | < 0.005 |
| Deviation from zero? | Significant | Significant | Significant |
| Equation | Y = -0.3577*X + 53.06 | Y = -3.935*X + 543.3 | Y = -5.167*X + 724.9 |
